# Supplementary material for: Host–Guest Cocrystallization of Phenanthrene[2]arene Macrocycles Facilitating Structure Determination of Liquid Organic Molecules
Source: Molecules. 2024 May 27;29(11):2523. doi: 10.3390/molecules29112523 (PMC11173633; doi:10.3390/molecules29112523)
Supplement: Supplementary file 1 [file molecules-29-02523-s001.zip › molecules-3005629-supplementary.pdf]

Supporting Information for

Host–Guest Cocrystallization of Phenanthrene[2]arene Macrocycles Facilitating Structure Determination of Liquid Organic Molecules

Guangchuan Ou, Yanfeng Zhang, Qiong Wang, Yingzhi Tan, Qiang Zhou and Fei Zeng

Table S1 Crystal data and structure refinement

Compound	**1**	**2**	**3**	**4**	**5**
Empirical formula	$C_{80}H_{74}Cl_2O_{12}$	$C_{73}H_{67}ClO_{12}$	$C_{80}H_{74}Br_2O_{12}$	$C_{90}H_{87}Cl_3O_{12}$	$C_{82}H_{78}Cl_2O_{12}$
Formula weight	1298.29	1171.71	1387.21	1466.94	1326.34
Temperature (K)	149.99(10)	149.99(10)	296.15	296.15	296.15
Crystal size (mm)	0.11×0.06× 0.03	0.23×0.08× 0.04	0.45×0.26× 0.20	0.20×0.16× 0.12	0.26×0.12× 0.10
Crystal system	Monoclinic	Monoclinic	Monoclinic	Monoclinic	triclinic
Space group	$P2_1/n$	$P2_1/n$	$P2_1/n$	$C2/c$	P-1
a / Å	13.8097(2)	15.0181(2)	13.892(15)	24.600(5)	9.685(6)
b / Å	12.1741(2)	8.1661(2)	12.235(13)	11.685(2)	12.064(8)
c / Å	19.8668(3)	26.5270(5)	20.123(2)	55.250(12)	16.632(10)
α / °	90	90	90	90	98.724(8)
β / °	94.6340(10)	104.861(2)	94.675(10)	102.86	94.198(8)
γ / °	90	90	90	90	97.049(8)
Volume /Å ^{3}	3329.10(9)	3144.43(11)	3393.7(6)	15483(6)	1898(2)
Z	2	2	2	8	1
D_c/g cm^{-3}	1.295	1.238	1.358	1.259	1.160
μ / mm^{-1}	1.404	1.049	1.259	0.182	0.144
F(000)	1368	1236	1440	6192	700
Reflections collected	26162	15257	37294	77415	21560
Unique refl. (R_{int})	6844 (0.0305)	6472 (0.0303)	7709(0.0371)	15911 (0.0405)	8476(0.0361)
Data/restraints/parameters	6844 /0 /431	6472 /233 /470	7709 /0 /431	15911 /813 /1126	8476/12 /440
Goodness-of-fit on F2	1.037	1.052	1.040	1.038	1.090
Final R indices [I >2σ (I)]	0.0486, 0.1349	0.0567, 0.1523	0.0662, 0.1889	0.0664, 0.1795	0.0686, 0.2144
R indices (all data)	0.0561, 0.1426	0.0669, 0.1633	0.1149, 0.2209	0.1187, 0.2084	0.1021, 0.2440
Largest diff. peak and hole /e.Å $^{-3}$	0.500 and -0.620	0.770 and -0.770	0.760 and -0.990	0.440 and -0.360	0.570 and -0.800

Compound	**6**	**7**	**8**	**9**	**10**
Empirical formula	$C_{82}H_{78}Cl_2O_{12}$	$C_{82}H_{78}Br_2O_{12}$	$C_{82}H_{78}Br_2O_{12}$	$C_{82}H_{78}Br_2O_{12}$	$C_{82}H_{78}Cl_2O_{12}$
Formula weight	1326.34	1415.26	1415.26	1415.26	1326.34
Temperature (K)	296.15	296.15	296.15	296.15	296.15
Crystal size (mm)	0.36×0.30× 0.15	0.13×0.12× 0.10	0.15×0.13× 0.12	0.25×0.15× 0.12	0.48×0.30× 0.25
Crystal system	Monoclinic	triclinic	Monoclinic	Monoclinic	Monoclinic
Space group	$P2_1/n$	P-1	$P2_1/n$	$P2_1/n$	$P2_1/n$
a / Å	13.8557(17)	9.529(18)	13.951(12)	14.060(12)	13.936(12)
b / Å	12.8723(16)	11.830(2)	12.444(11)	12.679(11)	12.592(11)
c / Å	19.500(3)	16.400(3)	19.384(17)	19.696(17)	19.471(16)
α / °	90	98.850(2)	90	90	90
β / °	91.377(2)	94.890(2)	92.211(12)	92.204(12)	92.012(10)
γ / °	90	96.760(2)	90	90	90
Volume /Å ^{3}	3476.8(8)	1804(6)	3363.0(5)	3508(5)	3414.8(5)
Z	2	1	2	2	2
D_c/g cm^{-3}	1.267	1.303	1.398	1.340	1.290
μ / mm^{-1}	0.157	1.185	1.272	1.219	0.160
F(000)	1400	736	1472.0	1472.0	1400
Reflections collected	36668	16679	28228	38407	37625
Unique refl. (R_{int})	7197(0.0361)	6408(0.1421)	5922(0.1043)	8057(0.0766)	7784 (0.0243)
Data/restraints/parameters	7197/652 /593	6408/288 /510	5922 /238 /481	8057 /0 /440	7784 /14/440
Goodness-of-fit on F2	1.043	0.842	1.092	1.045	1.076
Final R indices [I >2σ (I)]	0.0760, 0.2291	0.0889, 0.2211	0.1206, 0.3133	0.0702, 0.2011	0.0769, 0.2355
R indices (all data)	0.1173, 0.2692	0.2655, 0.3239	0.2547, 0.4066	0.1838, 0.2546	0.1044, 0.2619
Largest diff. peak and hole /e.Å $^{-3}$	0.410 and -0.450	0.390 and -0.500	0.430 and -0.600	0.520 and -0.790	0.910 and -0.680

Compound	**11**	**12**	**13**	**14**	**15**
Empirical formula	$C_{82}H_{78}Cl_2O_{12}$	$C_{82}H_{78}Cl_2O_{12}$	$C_{80}H_{74}Cl_2O_{12}$	$C_{80}H_{74}Br_2O_{12}$	$C_{76}H_{70}N_2O_{12}$
Formula weight	1326.34	1326.34	1298.29	1387.21	1203.34
Temperature (K)	296.15	296.15	296.15	296.15	296.15
Crystal size (mm)	0.42×0.26× 0.22	0.35×0.28× 0.15	0.36×0.18× 0.12	0.26×0.13× 0.12	0.15×0.13× 0.12
Crystal system	Monoclinic	Monoclinic	Monoclinic	Monoclinic	Monoclinic
Space group	$P2_1/n$	$P2_1/c$	$P2_1/n$	$P2_1/n$	$P2_1/n$
a / Å	14.005(3)	14.366(14)	13.986(9)	13.960(15)	14.869(11)
b / Å	12.492(2)	15.960(15)	12.450(8)	12.398(13)	8.353(6)
c / Å	19.455(3)	14.959(14)	19.559(12)	19.510(2)	26.77(2)
α / °	90	90	90	90	90
β / °	92.275(2)	97.483(13)	92.724(8)	92.673(2)	105.052(10)
γ / °	90	90	90	90	90
Volume /Å ^{3}	3401.1(10)	3401.0(6)	3402.0(4)	3373.3(6)	3211(4)
Z	2	2	2	2	2
D_c/g cm^{-3}	1.295	1.295	1.267	1.366	1.245
μ / mm^{-1}	0.161	0.161	0.159	1.266	0.084
F(000)	1400	1400	1368	1440	1272
Reflections collected	36569	38146	29116	37142	34768
Unique refl. (R_{int})	7727 (0.0428)	7851 (0.0872)	5787 (0.1085)	7663(0.0747)	7275 (0.0646)
Data/restraints/parameters	7727 /312/509	7851 /1/440	5787 /1 /430	7663 /48 /430	7275 /278 /506
Goodness-of-fit on F2	1.064	1.017	0.965	1.020	1.006
Final R indices [I >2σ (I)]	0.0729, 0.2266	0.0872, 0.2620	0.1015, 0.1843	0.0644, 0.1720	0.0720, 0.1927
R indices (all data)	0.0953, 0.2517	0.1792, 0.3268	0.1880, 0.2319	0.1620, 0.2179	0.1598, 0.2486
Largest diff. peak and hole /e.Å $^{-3}$	0.540 and -0.610	1.070 and -0.390	0.340 and -0.450	0.720 and -0.710	0.260 and -0.280

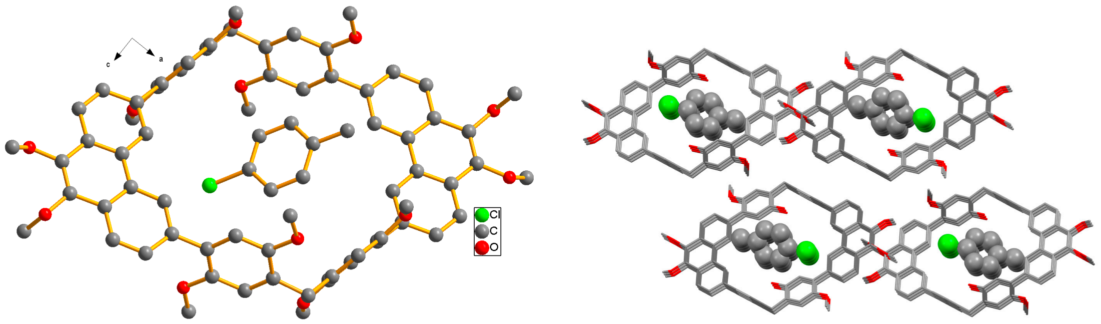

Figure S1. The structure of complex **2**, and the channels are filled with guest 1-chloro-4-methylbenzene molecules along the b axis.

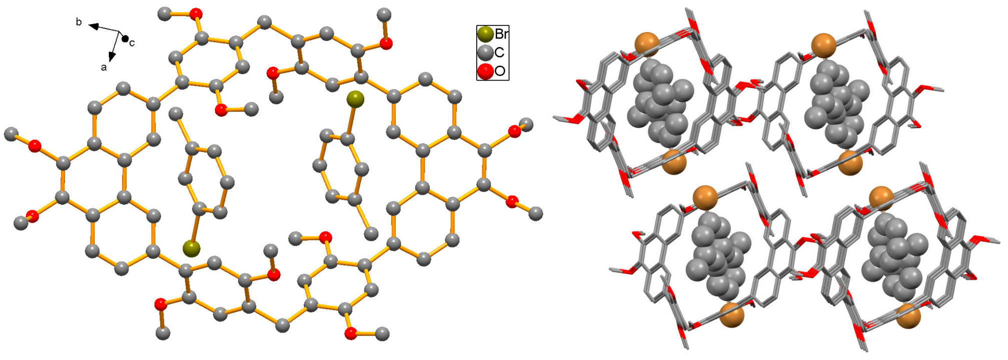

Figure S2. The structure of complex **3**, and the channels are filled with guest 1-bromo-3-methylbenzene molecules along the *b* axis.

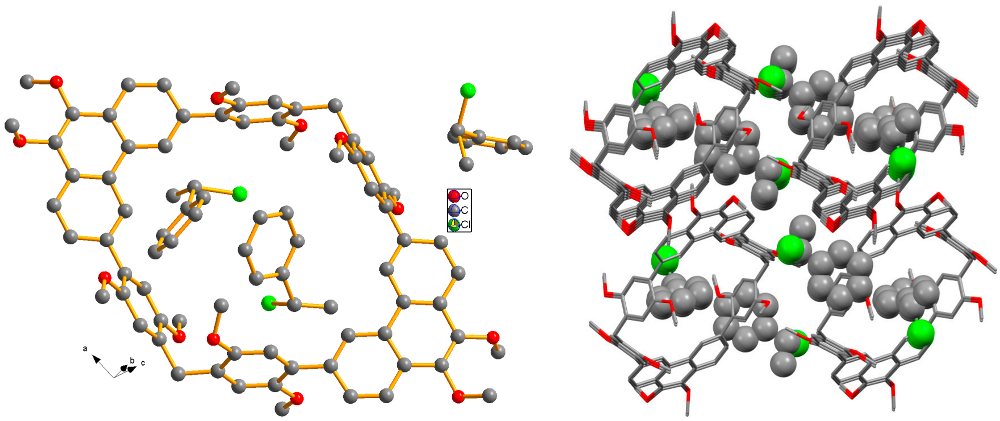

Figure S3. The structure of complex **4**, and the channels are filled with guest (1-chloroethyl)benzene molecules along the *b* axis.

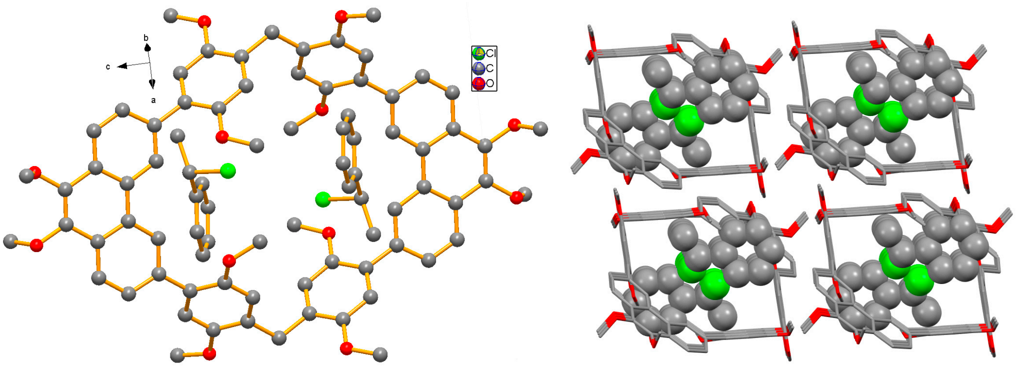

Figure S4. The structure of complex **5**, and the channels are filled with guest (*R*)-(1-chloroethyl)benzene molecules along the *b* axis.

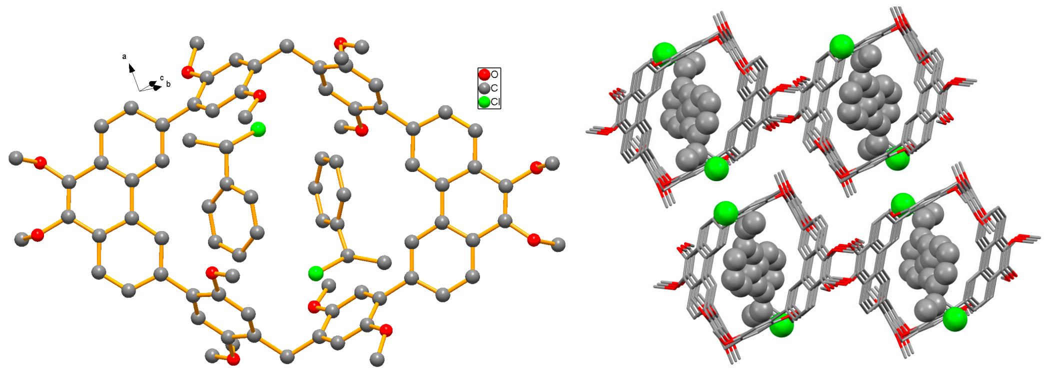

Figure S5. The structure of complex **6**, and the channels are filled with guest (*S*)-(1-chloroethyl)benzene molecules along the *b* axis.

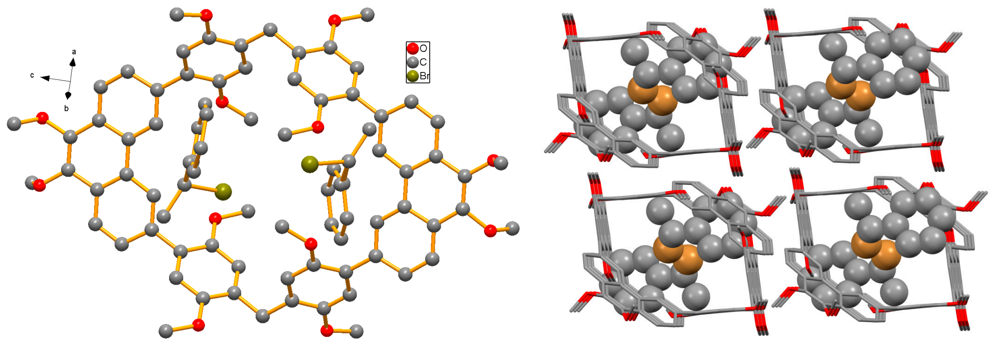

Figure S6. The structure of complex **7**, and the channels are filled with guest (1-bromoethyl)benzene molecules along the *b* axis.

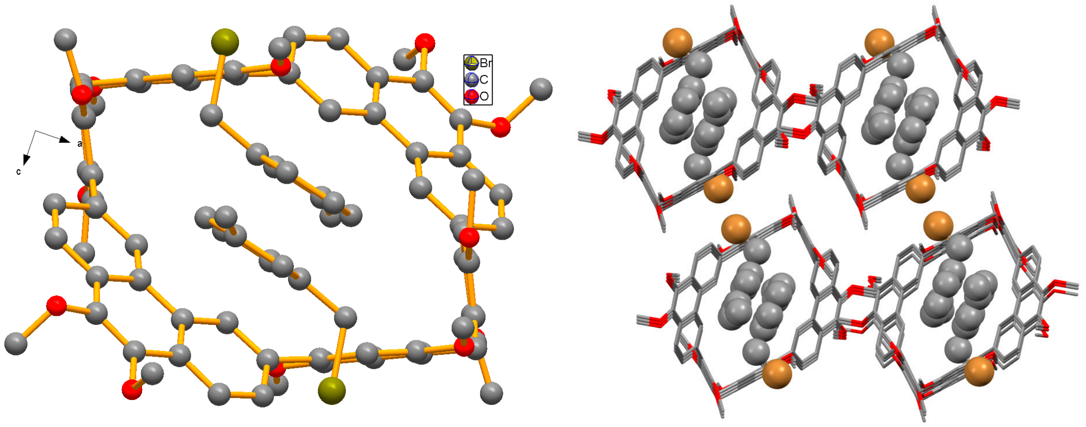

Figure S7. The structure of complex **8**, and the channels are filled with guest 1-(bromomethyl)-3-methylbenzene molecules along the *b* axis.

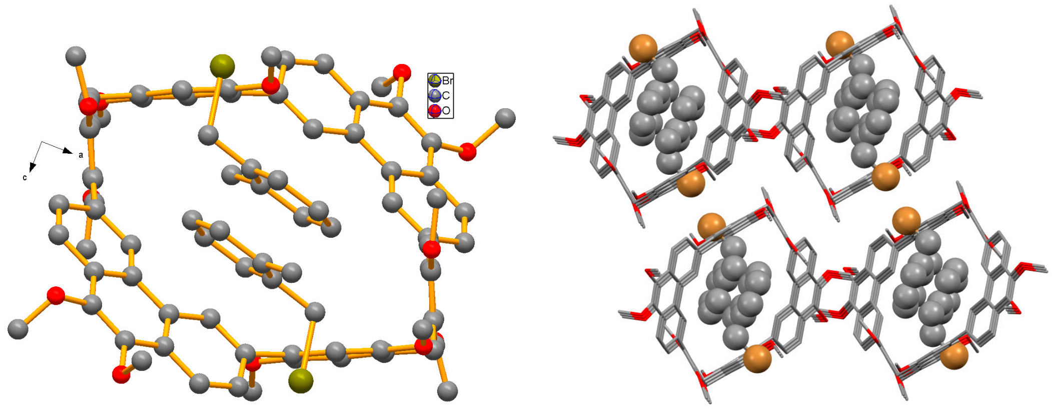

Figure S8. The structure of complex **9**, and the channels are filled with guest 1-(bromomethyl)-2-methylbenzene molecules along the *b* axis.

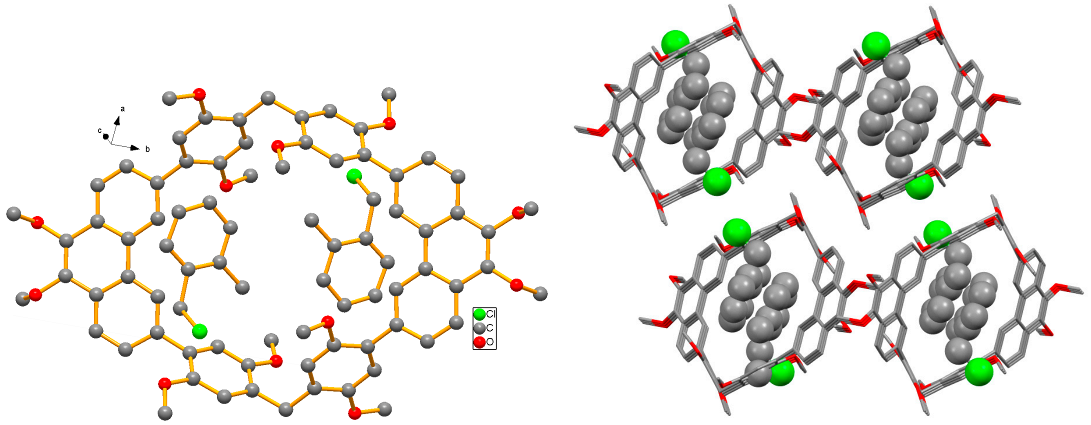

Figure S9. The structure of complex **10**, and the channels are filled with guest 1-(chloromethyl)-2-methylbenzene molecules along the *b* axis.

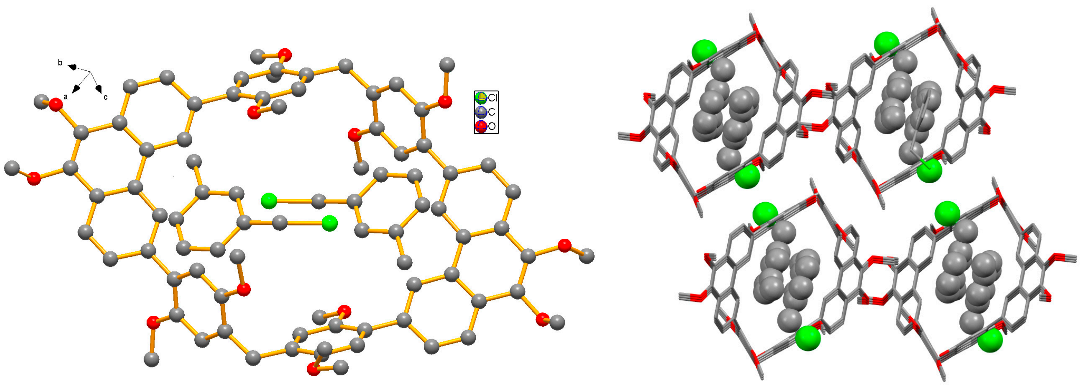

Figure S10. The structure of complex **11**, and the channels are filled with guest 1-(chloromethyl)-3-methylbenzene molecules along the *b* axis.

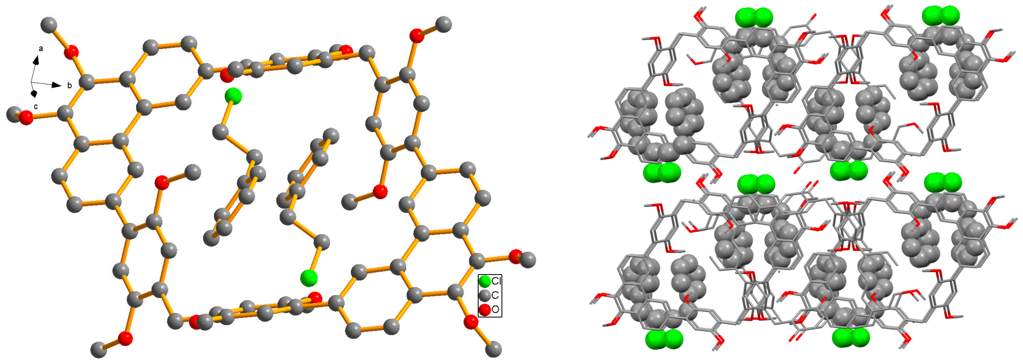

Figure S11. The structure of complex 12, and the channels are filled with guest (2-chloroethyl)benzene molecules along the *b* axis.

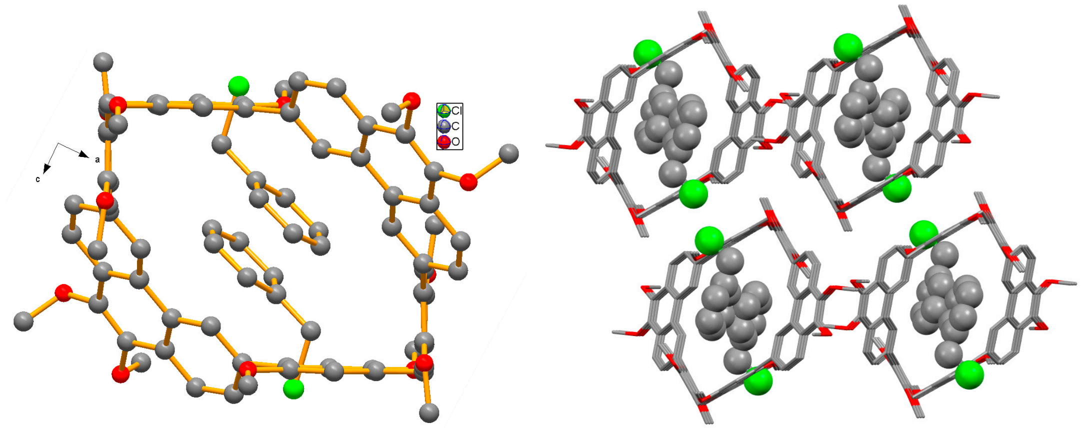

Figure S12. The structure of complex **13**, and the channels are filled with guest (chloromethyl)benzene molecules along the *b* axis.

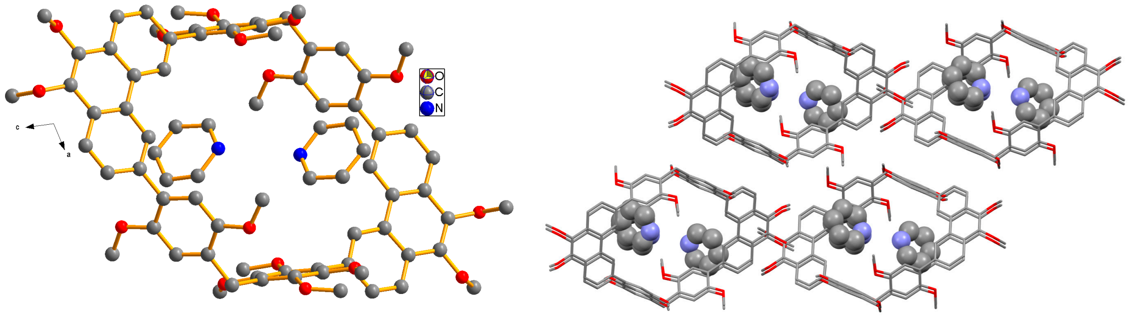

Figure S13. The structure of complex **15**, and the channels are filled with guest pyridine molecules along the *b* axis.
